# Impaired phonemic discrimination in logopenic variant primary progressive aphasia

**DOI:** 10.1002/acn3.51101

**Published:** 2020-06-18

**Authors:** Jeremy C. S. Johnson, Jessica Jiang, Rebecca L. Bond, Elia Benhamou, Maï‐Carmen Requena‐Komuro, Lucy L. Russell, Caroline Greaves, Annabel Nelson, Harri Sivasathiaseelan, Charles R. Marshall, Anna P. Volkmer, Jonathan D. Rohrer, Jason D. Warren, Chris J. D. Hardy

**Affiliations:** ^1^ Dementia Research Centre Department of Neurodegenerative Disease UCL Queen Square Institute of Neurology London UK; ^2^ Preventive Neurology Unit Wolfson Institute of Preventive Medicine Queen Mary University of London London UK

## Abstract

Logopenic variant primary progressive aphasia (lvPPA) is the least well defined of the major primary progressive aphasia (PPA) syndromes. We assessed phoneme discrimination in patients with PPA (semantic, nonfluent/agrammatic, and logopenic variants) and typical Alzheimer’s disease, relative to healthy age‐matched participants. The lvPPA group performed significantly worse than all other groups apart from tAD, after adjusting for auditory verbal working memory. In the combined PPA cohort, voxel‐based morphometry correlated phonemic discrimination score with grey matter in left angular gyrus. Our findings suggest that impaired phonemic discrimination may help differentiate lvPPA from other PPA subtypes, with important diagnostic and management implications.

## Introduction

The logopenic variant of primary progressive aphasia (lvPPA) is the least well defined of the three major primary progressive aphasia (PPA) syndromes.^1^ Whereas current diagnostic criteria for PPA emphasise impaired language output and linguistic processing,^2^ deficits of auditory analysis are increasingly recognised, particularly in lvPPA and nonfluent/agrammatic variant (nfv)PPA.^3^, ^4^, ^5^ These deficits remain poorly defined but may be particularly relevant to the representation of phonemes as “auditory objects” in lvPPA.^3^, ^6^, ^7^, ^8^ Here, we assessed phonemic discrimination and its neuroanatomical correlates in patients representing all major PPA variants, patients with typical Alzheimer’s disease (tAD) and healthy age‐matched individuals. Based on previous work,^2^, ^6^, ^9^ we predicted that phonemic discrimination would be most markedly affected in lvPPA, with a regional grey matter correlate in left temporo‐parietal cortex.

## Methods

### Participant characteristics

Eighty‐one patients (20 lvPPA, 24 nfvPPA, 22 svPPA, 15 tAD) were recruited from a larger longitudinal research cohort; all fulfilled relevant consensus diagnostic criteria and met study‐specific inclusion criteria (see Supplementary Material online). Seventy‐three healthy individuals also participated. Syndromic diagnoses were corroborated by a general neuropsychological assessment including measures of auditory verbal working memory (reverse digit span) and reading ability (assessed with National Adult Reading Test and Schonell Graded Word Reading Test) and with brain MRI (details in Supplementary Material). Participant characteristics are summarised in Table 1.

**Table 1:** Demographic, clinical, and neuropsychological data for participant groups

	lvPPA	nfvPPA	svPPA	tAD	Control	Omnibus comparison
N (M:F)	20 (6:14)	24 (16:8)	22 (5:17)	15 (3:12)	73 (41:32)	***P* = 0.001**
Age (years)	66.57 (7.73)	67.61 (8.89)	65.82 (6.85)	68.98 (5.92)	65.77 (7.28)	F(4,149) = 0.77, *P* = 0.545
Symptom duration (years)	4.77 (2.02)	4.28 (1.59)	6.14 (3.47)	5.91 (2.43)	‐	**χ^2^(3) = 9.73, *P* = 0.021**
WASI Matrices N (/32)	12.45 (7.34)	18.58 (7.17)	23.09 (7.28)	14.33 (8.88)	25.36 (6.62)^b^	**χ^2^ (4) = 55.13, *P* < 0.001**
Handedness (L:R)	2:18	5:19	2:20	1:14	8:43^c^	*P* = 0.736
Years of education	15.15 (2.37)	13.83 (2.65)	14.91 (3.29)	15.40 (3.22)	15.49 (2.78)^d^	χ^2^(4) = 7.59, *P* = 0.108
Hearing composite (dB)^a^	30.88 (8.14)	31.11 (9.99)	29.17 (9.89)	‐	25.18 (4.48)	χ^2^(3) = 5.90, *P* = 0.114
Reading score (IQ)	94.23 (19.10)	92.01 (20.48)	100.56 (15.06)	109.19 (10.76)	120.29 (5.18)	**χ^2^(4) = 78.86, *P* < 0.001**
Digit span forward (max digits)	3.90 (1.29)	5.08 (1.28)	7.14 (0.94)	5.80 (1.08)	6.86 (1.03)	**χ^2^(4) = 66.05, *P* < 0.001**
Digit span reverse (max digits)	2.70 (1.22)	3.35 (1.53)	5.27 (1.35)	3.87 (1.36)	5.19 (1.22)	**χ^2^(4) = 56.65, *P* < 0.001**
PALPA‐3 (/36)	31.80 (4.10)^e^	34.67 (1.71)	35.32 (1.17)	33.53 (1.68)^e^	35.64 (0.71)	**F(4,146) = 8.70, *P* < 0.001**

Mean (standard deviation) values are shown for continuous variables; distributions are shown for categorical variables. The right hand column gives results of relevant statistical omnibus tests (details in Methods); significant between‐group comparisons (*P* < 0.05) are in bold.

Abbreviations: Control, healthy control participant group; F, female; L, left; lvPPA, patient group with logopenic variant primary progressive aphasia; M, male; N, number; nfvPPA, patient group with nonfluent/agrammatic variant primary progressive aphasia; PALPA‐3, Psycholinguistic Assessments of Language Processing in Aphasia – Test 3 (see text for details); R, right; svPPA, patient group with semantic variant primary progressive aphasia; WASI, Wechsler Abbreviated Scale of Intelligence.

^a^ Hearing composite scores based on pure tone audiometry performance were available for a subset of each participant group (lvPPA n = 10; nfvPPA n = 9; svPPA n = 12; Control n = 28); no hearing data were available for tAD patients.

^b^ Datum was missing for one control participant.

^c^ Handedness data were not available for 22 healthy control participants.

^d^ Years of education were not recorded for eight healthy control participants.

^e^ Significantly worse performance versus healthy control group in model adjusting for auditory verbal working memory (reverse digit span), reading ability (reading IQ) and gender (*P* < 0.05). Fifty‐four potential participants failing to meet study‐specific inclusion criteria (the majority with a diagnosis of nfvPPA) were excluded from the study (details in Table S1).

All participants gave informed consent, in accordance with Declaration of Helsinki guidelines; ethical approval was granted by the UCL/UCLH Research Ethics Committees.

### Behavioural paradigm and analysis

Stimuli were selected from the Psycholinguistic Assessments of Language Processing in Aphasia (PALPA) battery “minimal pairs” Test 3 (PALPA‐3)^10^ which assesses discrimination of phonemes differing on a single acoustic characteristic. On each trial, participants must underline which of two written words matches a spoken monosyllabic word (e.g., spoken “leave” – written *leave/leaf*). We adopted a subset of 36 trials from the full PALPA‐3 test (Table S2). Pure tone audiometry data, available for 59 participants, were used to generate a composite measure of peripheral hearing function (procedural details in Supplementary Material online).

All data were analysed using Stata 14.0. The participant groups were compared on demographic and background neuropsychological data using ANOVAs for continuous variables and Fisher’s exact tests for categorical variables, with non‐parametric equivalents employed when assumptions of the general linear model were violated. Pearson’s correlations were used to assess the relationship between phonemic discrimination score and peripheral hearing score, and phonemic discrimination score and active auditory verbal working memory (maximum reverse digit span) in the combined patient cohort. An ANCOVA model was used to analyse participant group phonemic discrimination performance, with diagnosis as independent variable, PALPA‐3 score as dependent variable, and adjusting for reading IQ score, gender and reverse digit span. We compared the parametric ANCOVA with an alternative, non‐parametric approach allowing relaxation of normality and heteroscedasticity assumptions made by ANCOVA (details in Supplementary Material).

A threshold of *P* < 0.05 was accepted as the criterion for statistical significance throughout.

### Brain image acquisition and analysis

For the voxel‐based morphometry (VBM) analysis, PPA patients’ brain images were pre‐processed using SPM12 software, following previously described procedures^4^ (details in Supplementary Material). A multiple regression model was used to assess associations between voxel‐wise grey matter volume and PALPA‐3 score, adjusting for diagnosis, age, reverse digit span, and total intracranial volume. Statistical parametric maps were generated using an initial cluster‐forming threshold (*P* < 0.001) and assessed at peak statistical significance level *P* < 0.05 after family‐wise error (FWE) correction for multiple voxel‐wise comparisons within a pre‐specified anatomical region (Figure S1) based on previous work,^3^, ^9^ comprising left posterior superior temporal, supramarginal and angular gyri, and planum temporale.

## Results

### General participant group characteristics

The participant groups differed significantly (see Table 1) in gender (*P* = 0.001), mean symptom duration (χ^2^(3) = 9.73, *P* = 0.021), WASI Matrices score (an index of disease severity; χ^2^(4) = 55.13, *P* < 0.001), reading ability (χ^2^(4) = 78.86, *P* < 0.001), and digit span (forward, χ^2^(4) = 66.05, *P* < 0.001; reverse, χ^2^(4) = 56.65, *P* < 0.001). There were no other significant differences between participant groups (details in Supplementary Material online).

### Performance on phonemic discrimination

**Figure 1:**
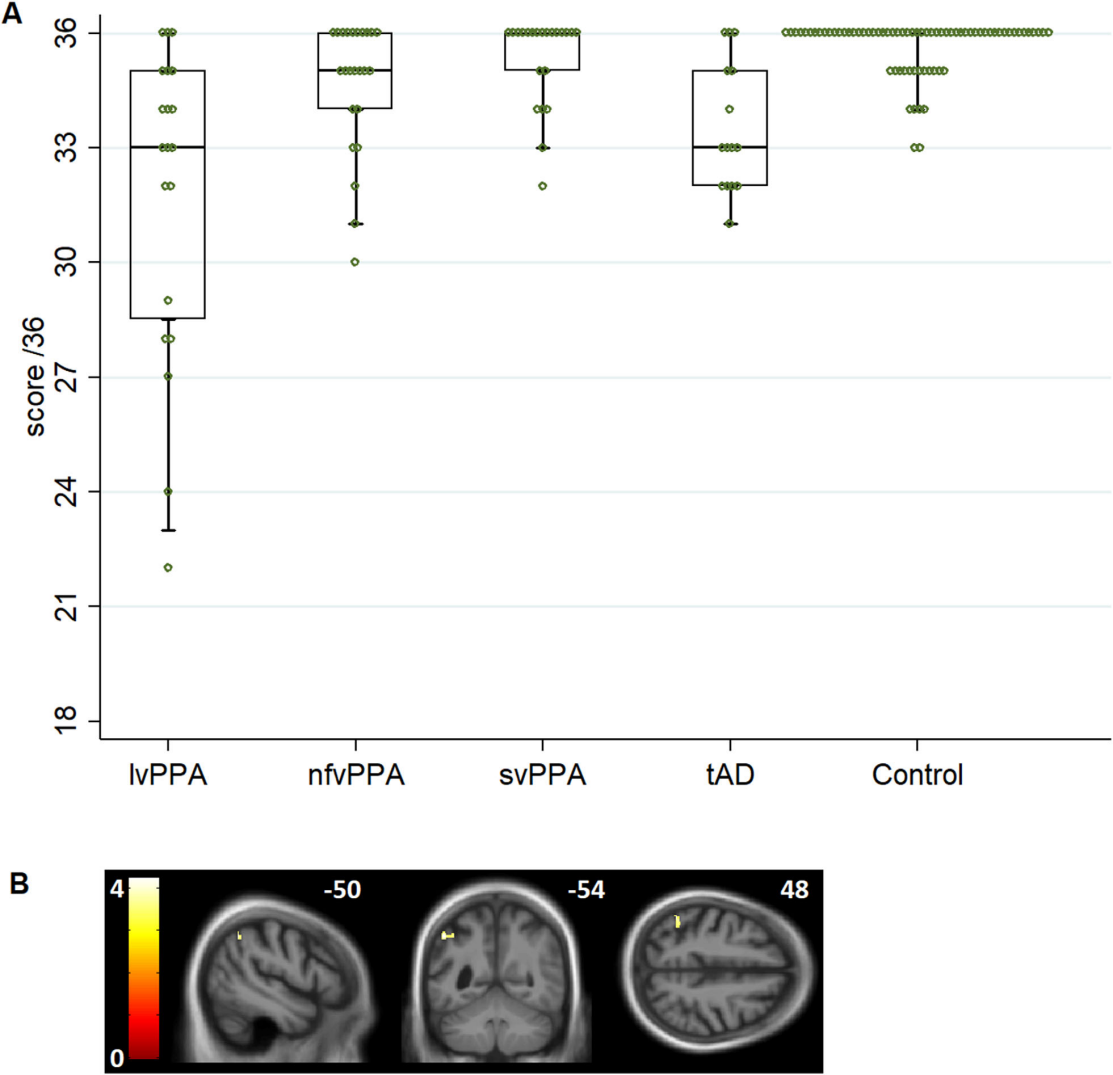
A, Profiles of participant group performance on the PALPA‐3 minimal pairs task (see also Table 1). Circles show individual participant performance. For each group, horizontal lines indicate median score, oblongs code interquartile range and whiskers 95% confidence intervals; a score of 18 would correspond to chance performance. lvPPA, patient group with logopenic variant primary progressive aphasia; nfvPPA, patient group with nonfluent/agrammatic variant primary progressive aphasia; svPPA, patient group with semantic variant primary progressive aphasia; tAD, patient group with typical Alzheimer’s disease. B, Statistical parametric maps showing regional grey matter in left angular gyrus positively associated with performance on the PALPA‐3 minimal pair discrimination task in the combined PPA patient cohort (n = 61). Maps are rendered on sagittal (left), coronal (middle) and axial (right) sections of the group mean T1‐weighted MR brain image in MNI space, thresholded at *P* < 0.001 uncorrected for multiple voxel‐wise comparisons over the whole brain for display purposes (the area indicated is significant at *P* = 0.031_FWE_ within the prespecified neuroanatomical region of interest (see Supplementary Material online). The colour bar indicates voxel‐wise t‐values, and the plane of each section is indicated using the corresponding MNI co‐ordinate.

The participant groups differed significantly in their performance on the PALPA‐3 task *F*(4,146) = 8.79, *P* < 0.001; see Table 1, Figure 1A). Post‐hoc comparisons between the groups revealed that this was driven by the lvPPA group performing significantly worse than the nfvPPA (t = −5.03, *P* < 0.001), svPPA (t = −4.64, *P* < 0.001), and healthy control (t = −3.98, *P* < 0.001) groups. The tAD group also performed significantly worse than the nfvPPA (t = −2.89, *P* = 0.004), svPPA (t = −2.99, *P* = 0.003), and healthy control (t = −2.48, *P* = 0.014) groups. No other group differences were significant. Including hearing composite score as an additional covariate in the model revealed a similar performance profile of the lvPPA group versus other participant groups (see Supplementary Material online). Comparison with a non‐parametric approach yielded similar results (details in Table S3).

Inspecting individual scores (Figure 1A), 40% (8/20) of patients in the lvPPA group scored below the healthy control range, compared to 12.5% (3/24) in the nfvPPA group, 4.5% (1/22) in the svPPA group, and 33.3% (5/15) in the tAD group. While the most severe individual phonemic discrimination deficits were exhibited by patients with lvPPA, there was wide variation in performance within groups.

### Correlations with working memory and hearing

PALPA‐3 score was significantly correlated both with reverse digit span across the entire cohort (r = 0.47, *P* < 0.001) and with hearing composite score in the subset of participants for whom hearing data were available (r = −0.278, *P* = 0.033).

### Neuroimaging associations

Across the PPA cohort, performance on the PALPA‐3 task was significantly positively associated with grey matter volume in left angular gyrus (t = 4.22, *P* = 0.031_FWE_). No other regional grey matter associations were identified.

## Discussion

Our findings demonstrate that patients with lvPPA (and to a lesser extent, tAD) perform worse on phonemic discrimination than both healthy older individuals and patients with other major variants of PPA. This deficit was not attributable to reduced auditory verbal working memory capacity or peripheral hearing loss. Phonemic discrimination performance across the PPA cohort was positively correlated with grey matter volume in left angular gyrus, a region that is likely to be core to the pathophysiology of lvPPA.^3^, ^6^, ^7^, ^8^

These findings corroborate previous work showing that patients with lvPPA perform poorly on tasks requiring manipulation of phonemic representations (e.g., phoneme deletion tasks^6^) or decoding of phonemic spectrotemporal features.^3^ Phonemic discrimination relies on fine‐grained analysis of the “boundaries” that define phonemes as auditory objects: it could therefore be considered to probe the auditory and linguistic processing interface, an earlier processing stage than is conventionally assessed in the linguistic evaluation of PPA. Categorical representations of phonemes that are normally sharply defined^11^ might plausibly become “blurred” in lvPPA, making fine‐grained phonemic discriminations more difficult. This work builds on previous evidence that PPA syndromes have specific profiles of auditory cognitive dysfunction.^3^, ^5^

Angular gyrus in the dominant hemisphere is targeted in lvPPA,^7^ and has previously been implicated in disambiguating degraded speech signals in PPA and tAD^4^ and in categorical phoneme discrimination in healthy participants.^12^ This region is affected in different variants of AD,^1^, ^7^ providing a candidate neural substrate for the similar profiles of impaired phonemic discrimination in the lvPPA and tAD groups, although we note neuroanatomical associations were only assessed in the PPA cohort here. Individual patients with lvPPA were not all impaired on this task, consistent with previous evidence that phonologic errors are not produced by every patient with lvPPA.^8^ This suggests that phonemic processing deficits may stratify sub‐syndromes within lvPPA and raises the further possibility that deficits of phonemic perception and production may be coupled via fronto‐parietal processing streams.^13^ Although the present findings do not speak to this issue directly, the correlation between phoneme discrimination and reverse digit span (which requires repetition of a heard phoneme string) could potentially indicate a linkage between the accuracy of phonological input processing and speech output that could be explored in future work.

We regard this work as preliminary: in particular, its clinical relevance needs to be further substantiated. However, our findings foreground several key points of potential clinical relevance while suggesting opportunities for future work. PPA syndromes are often challenging to differentiate, even for experts; phoneme discrimination may further this differentiation, pending replication in larger patient cohorts. A key issue is individual variation and heterogeneity within PPA syndromes (Figure 1A), particularly nfvPPA (moreover, here we excluded those nfvPPA patients with the most severe speech production deficits). Speech perception deficits may go undetected unless objectively assessed, contributing significant concealed morbidity; on the other hand, patients who complain of poor speech perception may be offered inappropriate hearing amplification interventions, delaying potential benefit from speech and language therapy.

How phonemic discrimination relates both to phonological production during speech and to other aspects of nonverbal auditory perception in lvPPA should be clarified, both behaviourally and with neuroimaging techniques that can assess the structural and functional integrity of language networks. An exciting avenue would be to investigate whether phoneme discrimination can be ameliorated or maintained. Previous work has shown retained capacity for perceptual learning of degraded speech in lvPPA,^4^ and minimal pair discrimination training has been shown to improve auditory discrimination in the context of stroke aphasia.^14^ Minimal pair discrimination training in healthy second‐language learners benefits not only phonologic perception but also speech production,^15^ suggesting a novel, physiologically motivated strategy for “re‐tuning” phonological output in lvPPA.

## Author Contributions

JCSJ, JDW, and CJDH contributed to the conception and design of the study. JCSJ, CJDH, RLB, EB, M‐CR‐K, LLR, CG, AN, HS, CRM, APV, and JDR contributed to acquisition and analysis of data. JCSJ, JDW, and CJDH contributed to drafting of manuscript and figures.

## Conflicts of Interest

Nothing to report.

## Funding Information

No funding information provided.

## Supporting information


**Figure S1.** Neuroanatomical region of interest specified for VBM analysis. Representative coronal (top left), sagittal (top right), and axial (bottom) T1‐weighted MRI brain sections showing the neuroanatomical region (delineated in yellow) used to correct for multiple voxel‐wise comparisons in the voxel‐based morphometric (VBM) analysis, based on prior anatomical hypotheses (see text). This region comprised posterior superior temporal gyrus, supramarginal gyrus, angular gyrus, and planum temporale, all in the left hemisphere.

**Table S1.** Details of excluded cases, by participant group. The table shows details of potential participants excluded for not meeting inclusion criteria for this study. lvPPA, patient group with logopenic variant primary progressive aphasia; nfvPPA, patient group with nonfluent/agrammatic variant primary progressive aphasia; svPPA, patient group with semantic variant primary progressive aphasia; tAD, patient group with typical Alzheimer’s disease.

**Table S2.** Subset of items from original 72‐item PALPA‐3 test used in the experiment. The table gives the 36 pairs that were used in the present study. *Frequency* of the target (compared with the distractor) was manipulated in the original PALPA‐3: for half of the items the target has a higher frequency than the distractor; for the other half the target is lower or equivalent in frequency to the distractor. *Location* refers to the fact that pairs differ either in the initial or final positions of pairs, or in pairs that are metathetically related (i.e., the order of sounds is reversed). *Type* indicates whether the foil minimally deviates from the target in terms of voice, manner, or place of articulation.

**Table S3.** Comparison of original ANCOVA and adjusted model with relaxed normality assumptions. The main manuscript reports results from a parametric ANCOVA model. This table shows 95% confidence intervals (CIs) for between‐group comparisons for the conventional ANCOVA approach, compared to non‐parametric bias corrected and accelerated bootstrap confidence intervals for the between‐group differences based on 10000 bootstrap resamples, relaxing assumptions of normality and homoscedasticity. Results from this more conservative approach were very similar to those using the conventional ANCOVA, and in particular the same significant group differences (in bold) were yielded using both approaches.

**File S1.** Supplementary Methods and Results: Impaired phonemic discrimination in logopenic variant primary progressive aphasia.
