# Supplementary figures and images for: Impaired phonemic discrimination in logopenic variant primary progressive aphasia
Source: Ann Clin Transl Neurol. 2020 Jun 18;7(7):1252–7. doi: 10.1002/acn3.51101 (PMC7359108; doi:10.1002/acn3.51101)

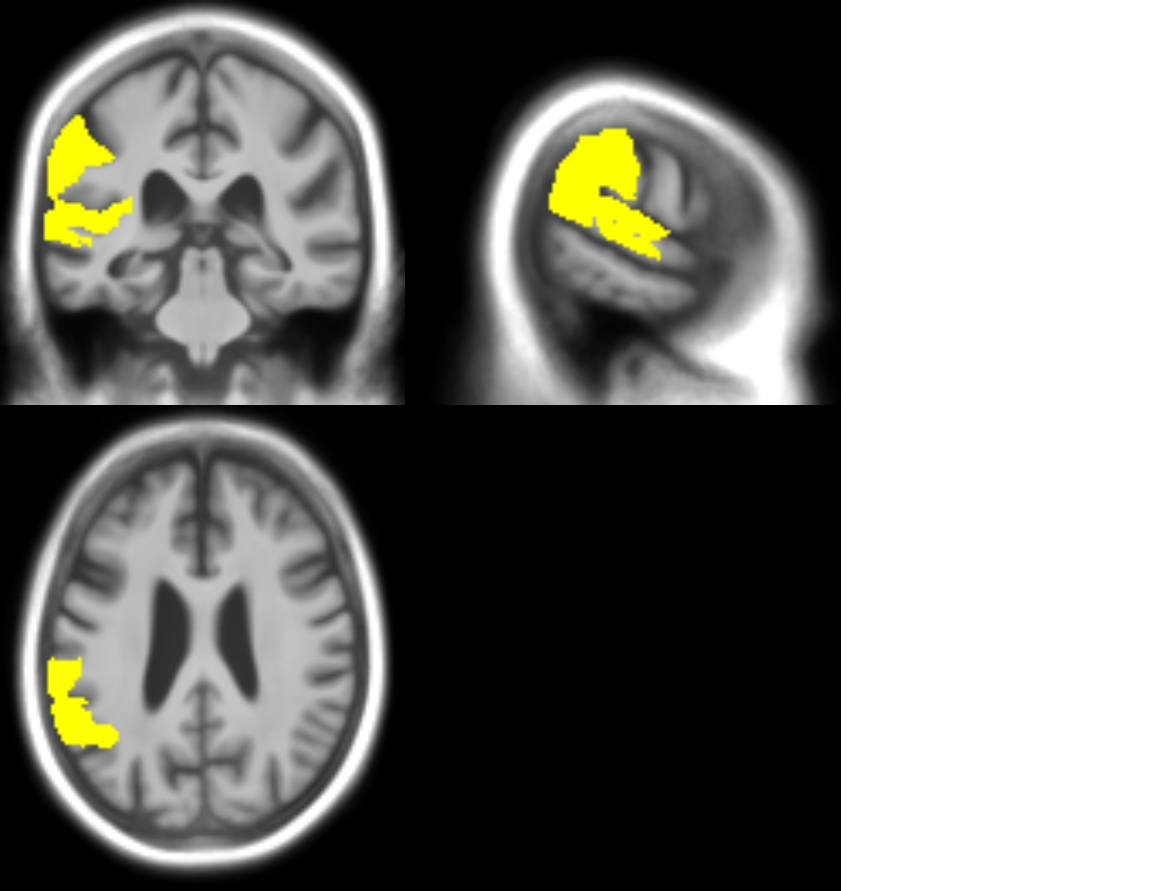

Supplement: Supplementary file 1 — Figure S1. Neuroanatomical region of interest specified for VBM analysis. Representative coronal (top left), sagittal (top right), and axial (bottom) T1‐weighted MRI brain sections showing the neuroanatomical region (delineated in yellow) used to correct for multiple voxel‐wise comparisons in the voxel‐based morphometric (VBM) analysis, based on prior anatomical hypotheses (see text). This region comprised posterior superior temporal gyrus, supramarginal gyrus, angular gyrus, and planum temporale, all in the left hemisphere. [file ACN3-7-1252-s001.tif]
