# Supplementary material for: Impaired phonemic discrimination in logopenic variant primary progressive aphasia
Source: Ann Clin Transl Neurol. 2020 Jun 18;7(7):1252–7. doi: 10.1002/acn3.51101 (PMC7359108; doi:10.1002/acn3.51101)
Supplement: Supplementary file 2 — Table S1. Details of excluded cases, by participant group. The table shows details of potential participants excluded for not meeting inclusion criteria for this study. lvPPA, patient group with logopenic variant primary progressive aphasia; nfvPPA, patient group with nonfluent/agrammatic variant primary progressive aphasia; svPPA, patient group with semantic variant primary progressive aphasia; tAD, patient group with typical Alzheimer’s disease. [file ACN3-7-1252-s002.docx]

**Table S1.** Details of excluded cases, by participant group

|  | **lvPPA** | **nfvPPA** | **svPPA** | **tAD** | **Control** |
| --- | --- | --- | --- | --- | --- |
| Hearing aid users | 0 | 1 | 1 | 0 | 1 |
| Non-native English speakers | 0 | 1 | 3 | 0 | 1 |
| Didn’t meet reading criterion | 1 | 3 | 4 | 1 | 0 |
| Unable to speak (so no reading score) | 0 | 7 | 0 | 0 | 0 |
| Didn’t understand PALPA-3 instructions | 3 | 5 | 0 | 0 | 0 |
| Presence of pathogenic mutation | 1 | 2 | 1 | 0 | 0 |
| Missing reading data | 2 | 5 | 3 | 0 | 0 |
| Missing data on age of onset or gender | 1 | 0 | 0 | 0 | 7 |
| Total | 8 | 24 | 12 | 1 | 9 |

The table shows details of potential participants excluded for not meeting inclusion criteria for this study. lvPPA, patient group with logopenic variant primary progressive aphasia; nfvPPA, patient group with nonfluent/agrammatic variant primary progressive aphasia; svPPA, patient group with semantic variant primary progressive aphasia; tAD, patient group with typical Alzheimer’s disease.
