# Supplementary material for: Impaired phonemic discrimination in logopenic variant primary progressive aphasia
Source: Ann Clin Transl Neurol. 2020 Jun 18;7(7):1252–7. doi: 10.1002/acn3.51101 (PMC7359108; doi:10.1002/acn3.51101)
Supplement: Supplementary file 3 — Table S2. Subset of items from original 72‐item PALPA‐3 test used in the experiment. The table gives the 36 pairs that were used in the present study. Frequency of the target (compared with the distractor) was manipulated in the original PALPA‐3: for half of the items the target has a higher frequency than the distractor; for the other half the target is lower or equivalent in frequency to the distractor. Location refers to the fact that pairs differ either in the initial or final positions of pairs, or in pairs that are metathetically related (i.e., the order of sounds is reversed). Type indicates whether the foil minimally deviates from the target in terms of voice, manner, or place of articulation. [file ACN3-7-1252-s003.docx]

**Table S2.** Subset of items from original 72-item PALPA-3 test used in the experiment.

| **No.** | **No. in original test** | **Target** | **Foil** | **Freq** | **Location** | **Type** |
| --- | --- | --- | --- | --- | --- | --- |
| **8** | 8 | Fall | Fawn | High | Final | Manner |
| **11** | 11 | Pain | Pail | High | Final | Manner |
| **24** | 29 | Cup | Cut | High | Final | Place |
| **26** | 32 | Run | Rung | High | Final | Place |
| **20** | 23 | Pig | Pick | High | Final | Voice |
| **28** | 38 | Code | Coat | High | Final | Voice |
| **4** | 4 | Bone | Moan | High | Initial | Manner |
| **23** | 28 | Sack | Tack | High | Initial | Manner |
| **7** | 7 | Deed | Bead | High | Initial | Place |
| **15** | 16 | Fail | Veil | High | Initial | Voice |
| **17** | 19 | Cut | Gut | High | Initial | Voice |
| **29** | 43 | Meat | Neat | High | Initial | Voice |
| **16** | 18 | Nod | Don | High | Metathetic | Manner |
| **34** | 57 | Lean | Kneel | High | Metathetic | Manner |
| **9** | 9 | Tape | Pate | High | Metathetic | Place |
| **12** | 12 | Moan | Gnome | High | Metathetic | Place |
| **14** | 15 | Toad | Dote | High | Metathetic | Voice |
| **36** | 72 | Debt | Ted | High | Metathetic | Voice |
| **5** | 5 | Rice | Write | Low | Final | Manner |
| **22** | 26 | Hen | Head | Low | Final | Manner |
| **32** | 50 | Robe | Road | Low | Final | Place |
| **2** | 2 | Leave | Leaf | Low | Final | Voice |
| **18** | 20 | Live | Life | Low | Final | Voice |
| **30** | 47 | Fang | Fan | Low | Final | Voice |
| **10** | 10 | Nip | Lip | Low | Initial | Manner |
| **25** | 31 | Mat | Bat | Low | Initial | Manner |
| **13** | 13 | Feed | Seed | Low | Initial | Place |
| **19** | 22 | Down | Gown | Low | Initial | Place |
| **1** | 1 | Pill | Bill | Low | Initial | Voice |
| **27** | 34 | Dale | Tale | Low | Initial | Voice |
| **6** | 6 | Mob | Bomb | Low | Metathetic | Manner |
| **31** | 48 | Nail | Lane | Low | Metathetic | Manner |
| **3** | 3 | Pat | Tap | Low | Metathetic | Place |
| **21** | 24 | Dab | Bad | Low | Metathetic | Place |
| **33** | 54 | Tuck | Cut | Low | Metathetic | Place |
| **35** | 66 | Mane | Name | Low | Metathetic | Place |

The table gives the 36 pairs that were used in the present study. *Frequency* of the target (compared with the distractor) was manipulated in the original PALPA-3: for half of the items the target has a higher frequency than the distractor; for the other half the target is lower or equivalent in frequency to the distractor. *Location* refers to the fact that pairs differ either in the initial or final positions of pairs, or in pairs that are metathetically related (i.e. the order of sounds is reversed). *Type* indicates whether the foil minimally deviates from the target in terms of voice, manner, or place of articulation.
