# Supplementary material for: Impaired phonemic discrimination in logopenic variant primary progressive aphasia
Source: Ann Clin Transl Neurol. 2020 Jun 18;7(7):1252–7. doi: 10.1002/acn3.51101 (PMC7359108; doi:10.1002/acn3.51101)
Supplement: Supplementary file 4 — Table S3. Comparison of original ANCOVA and adjusted model with relaxed normality assumptions. The main manuscript reports results from a parametric ANCOVA model. This table shows 95% confidence intervals (CIs) for between‐group comparisons for the conventional ANCOVA approach, compared to non‐parametric bias corrected and accelerated bootstrap confidence intervals for the between‐group differences based on 10000 bootstrap resamples, relaxing assumptions of normality and homoscedasticity. Results from this more conservative approach were very similar to those using the conventional ANCOVA, and in particular the same significant group differences (in bold) were yielded using both approaches. [file ACN3-7-1252-s004.docx]

**Table S3.** Comparison of original ANCOVA and adjusted model with relaxed normality assumptions

|  | Coefficient | Original ANCOVA  (95% CIs) | Adjusted ANCOVA (95% CIs) |
| --- | --- | --- | --- |
| lvPPA vs nfvPPA | **-2.73** | -3.80 to -1.66 | -4.60 to -1.10 |
| lvPPA vs svPPA | **-2.80** | -3.99 to -1.61 | -4.48 to -1.39 |
| lvPPA vs tAD | -0.96 | -2.18 to 0.26 | -2.77 to 0.54 |
| lvPPA vs Control | **-2.29** | -3.42 to -1.15 | -3.96 to -0.93 |
| tAD vs nfvPPA | **-1.77** | -2.97 to -0.56 | -2.98 to -0.48 |
| tAD vs svPPA | **-1.84** | -3.06 to -0.62 | -2.89 to -0.77 |
| tAD vs Control | **-1.33** | -3.82 to -0.27 | -2.27 to -0.18 |
| nfvPPA vs svPPA | -0.07 | -1.19 to 1.04 | -1.18 to 1.07 |
| nfvPPA vs Control | -0.44 | -0.60 to 1.48 | -0.68 to 1.80 |
| svPPA vs Control | -0.51 | -1.49 to 0.46 | -1.51 to 0.25 |

The main manuscript reports results from a parametric ANCOVA model. This table shows 95% confidence intervals (CIs) for between-group comparisons for the conventional ANCOVA approach, compared to non-parametric bias corrected and accelerated bootstrap confidence intervals for the between-group differences based on 10000 bootstrap resamples, relaxing assumptions of normality and homoscedasticity. Results from this more conservative approach were very similar to those using the conventional ANCOVA, and in particular the same significant group differences (in bold) were yielded using both approaches.
