# Supplementary material for: Impaired phonemic discrimination in logopenic variant primary progressive aphasia
Source: Ann Clin Transl Neurol. 2020 Jun 18;7(7):1252–7. doi: 10.1002/acn3.51101 (PMC7359108; doi:10.1002/acn3.51101)
Supplement: Supplementary file 5 — File S1. Supplementary Methods and Results: Impaired phonemic discrimination in logopenic variant primary progressive aphasia. [file ACN3-7-1252-s005.docx]

**SUPPLEMENTARY MATERIAL: Impaired phonemic discrimination in logopenic variant primary progressive aphasia, by JCS Johnson et al**

**METHODS**

**Study inclusion criteria**

No participant had a clinical history of primary otological disease, and hearing aid users were excluded. None had radiological evidence of significant co-morbid cerebrovascular disease. All participants were native English speakers. Successful completion of the main experimental task requires the participant to be able to read monosyllabic words successfully, so participants either had to score >14 on the difficult National Adult Reading Test^1^ or >18 on the Schonell Graded Word Reading Test^2^ (selected as a cut-off because the Schonell test contains 19 monosyllabic words). These were converted using formulae listed in the NART Second Edition test manual into a standard score that is intended to give an indication of premorbid IQ; we used these scores as indicators of general reading ability (‘reading IQ’). A total of 54 potential participants were excluded for not meeting the study-specific criteria (details in Table S1). All patients fulfilled relevant consensus diagnostic criteria^3,4^.

**Audiometry procedure**

As an index of peripheral hearing function, pure tone audiometry was performed using an Otovation Roto audiometer (www.otovation.com) in a quiet room using a standard clinical audiometry protocol^5^. Four frequency levels were tested (500, 1000, 2000, 4000 Hz). At each frequency, the participant was played three tones, starting at 20dB. If the participant indicated correctly that they had heard at least two of the three tones, this was recorded as the threshold for that frequency; if not, the level was increased in increments of 5dB up to 70dB. Hearing was assessed in both ears for each participant. The mean average across the four frequencies was calculated for each ear separately, and the lowest of these (i.e. reflecting the better ear) was used as a ‘peripheral hearing composite’ in analyses.

**Brain image acquisition and pre-processing**

Patients scanned before March 2016 were scanned using a 3.0T Siemen’s Trio MRI scanner using a 32-channel passed array head-coil and a T1-weighted sagittal 3D magnetization rapid gradient echo sequence (TE = 2.9 ms, TR = 900 ms, TI = 2200 ms), with dimensions of 256×256×208, and voxel size of 1.1×1.1×1.1 mm. Patients scanned after March 2016 were scanned using a 3.0T MAGNETOM Prisma scanner using a 64-channel head and-neck receiver array coil and a T1-weighted sagittal 3D magnetization-prepared rapid gradient-echo sequence (TE = 2.9 ms, TR = 850 ms, TI = 2000 ms), with matrix size 256 × 256 × 208 and voxel dimensions 1.1 × 1.1 × 1.1 mm.

For the VBM analysis, patients’ brain images were segmented, pre-processed and normalised to Montreal Neurological Institute (MNI) space with isotropic voxel size 1.5mm using SPM12 software (<http://www.fil.ion.ucl.ac.uk/som/software/spm12>) and the Diffeomorphic Anatomical Registration Through Exponentiated Lie Algebra (DARTEL) toolbox with default parameters in MATLAB R2013b (MathWarks, Natick, MA, USA). Images were smoothed using a 6mm full-width at half-maximum (FWHM) Gaussian kernel. To control for individual differences in brain size, total intracranial volume was calculated for each participant by summing the white matter, grey matter and cerebrospinal fluid volumes generated during the segmentation phase. A study-specific average brain was created by warping all patients’ native-space whole brain images to the final DARTEL template and using the ImCalc function to generate an average of these images: statistical parametric maps were overlayed on this image. For the analysis, we used an explicit mask generated via an automatic mask-creation strategy^6^.

**Analysis relaxing normality assumptions.**

The main manuscript reports results from parametric ANCOVA models. To check whether results with this approach were affected by violation of the assumption of normality, we ran a separate analysis in which we adopted a permutation approach attributable to Freedman and Lane^7^. This approach gives the same parameter estimates as ANCOVA but with p-values relaxing the normality and homoscedasticity assumptions made by ANCOVA. We also calculated non-parametric bias corrected and accelerated bootstrap confidence intervals^8^ for the between group differences based on 10000 bootstrap resamples; these also relax assumptions of normality and homoscedasticity.

**RESULTS**

**General participant group characteristics**

Participant groups differed significantly in gender distribution, Fisher exact p = 0.001, which was driven by the higher proportion of females to males in the nfvPPA group than the other three participant groups (Table 1). The non-parametric Kruskal-Wallis test suggested a significant difference between patient groups in mean symptom duration (χ^2^(3) = 9.73; p = 0.021), driven by a shorter duration in nfvPPA relative to tAD (z = -2.51, p = 0.012) and svPPA (z = -2.35, p = 0.019). Disease severity as indexed by WASI Matrices score was significantly different between participant groups (χ^2^(4)=55.13;p<0.001) with lvPPA worse than nfvPPA (z = -2.56, p = 0.014), lvPPA worse than svPPA (z = -3.94, p <0.001), no difference between lvPPA and tAD (z = -0.70, p = 0.483), nfvPPA worse than svPPA (z = -2.45, p = 0.014)and tAD worse than svPPA (z = -3.03, p = 0.003); and lvPPA (z = -5.73, p <0.001), nfvPPA (z = -4.31, p <0.001) and tAD (z = -4.65, p <0.001) all worse than healthy controls, but no difference between svPPA and healthy controls (z = -1.28, p =0.201).

There was a significant difference across groups in reading ability, χ^2^(4) = 78.86; p < 0.001, accounted for by the healthy control group performing better than all patient groups (all p < 0.001) and the tAD group scoring higher than patients with lvPPA (z = 2.22, p = 0.027) and nfvPPA (z = 2.56, p = 0.011). Forward digit span task performance also differed significantly across participant groups, χ^2^(4) = 78.86; p < 0.001, with the lvPPA group performing worse than each other participant group (all p <0.05), patients with nfvPPA performing worse than patients with svPPA (z = -4.66, p < 0.001) and Controls (z = -5.27, p < 0.001), and patients with tAD performing worse than patients with svPPA (z = -3.34, p = 0.001) and healthy controls (z = -3.21, p = 0.001). Similar patterns were seen for reverse digit span task performance, with an overall significant difference across participant groups, χ^2^(4) = 56.65; p < 0.001 explained by the lvPPA group performing significantly worse than all other groups (all p < 0.05), and the healthy control and svPPA groups both performing significantly better than the nfvPPA and tAD participant groups (p < 0.05).

There were no significant differences between groups in mean age [F(4,149) = 0.77, p = 0.545], handedness (p = 0.736), years of education [χ^2^(4) = 7.59; p = 0.108], or hearing score [χ^2^(3) = 5.90; p = 0.114]; see Table 1.

**Analysis relaxing normality assumptions**

The overall effect of diagnosis computed using 10000 permutations of residuals from a model adjusting for reading IQ score, gender and reverse digit span was p < 0.001. The non-parametric bias corrected and accelerated bootstrap confidence intervals were very similar to those using the conventional ANCOVA model (Table S3).

**Group profiles of phonemic discrimination performance: effect of adjusting for peripheral hearing function**

Running a regression model including hearing composite score as a covariate in addition to reading IQ score, gender and reverse digit span revealed a significant difference between groups (F(7,51)=5.98, p<0.001) driven by the lvPPA group performing significantly worse than the other three participant groups (vs nfvPPA t = 2.77, p = 0.008; vs svPPA t = 2.29, p = 0.026; vs Control t = 2.04, p = 0.047); no other between-group comparisons were significant. The tAD group was not included in this analysis as audiometry data were not available for this group.

**Table S1.** Details of excluded cases, by participant group

|  | **lvPPA** | **nfvPPA** | **svPPA** | **tAD** | **Control** |
| --- | --- | --- | --- | --- | --- |
| Hearing aid users | 0 | 1 | 1 | 0 | 1 |
| Non-native English speakers | 0 | 1 | 3 | 0 | 1 |
| Didn’t meet reading criterion | 1 | 3 | 4 | 1 | 0 |
| Unable to speak (so no reading score) | 0 | 7 | 0 | 0 | 0 |
| Didn’t understand PALPA-3 instructions | 3 | 5 | 0 | 0 | 0 |
| Presence of pathogenic mutation | 1 | 2 | 1 | 0 | 0 |
| Missing reading data | 2 | 5 | 3 | 0 | 0 |
| Missing data on age of onset or gender | 1 | 0 | 0 | 0 | 7 |
| Total | 8 | 24 | 12 | 1 | 9 |

The table shows details of potential participants excluded for not meeting inclusion criteria for this study. lvPPA, patient group with logopenic variant primary progressive aphasia; nfvPPA, patient group with nonfluent/agrammatic variant primary progressive aphasia; svPPA, patient group with semantic variant primary progressive aphasia; tAD, patient group with typical Alzheimer’s disease.

**Table S2.** Subset of items from original 72-item PALPA-3 test used in the experiment.

| **No.** | **No. in original test** | **Target** | **Foil** | **Freq** | **Location** | **Type** |
| --- | --- | --- | --- | --- | --- | --- |
| **8** | 8 | Fall | Fawn | High | Final | Manner |
| **11** | 11 | Pain | Pail | High | Final | Manner |
| **24** | 29 | Cup | Cut | High | Final | Place |
| **26** | 32 | Run | Rung | High | Final | Place |
| **20** | 23 | Pig | Pick | High | Final | Voice |
| **28** | 38 | Code | Coat | High | Final | Voice |
| **4** | 4 | Bone | Moan | High | Initial | Manner |
| **23** | 28 | Sack | Tack | High | Initial | Manner |
| **7** | 7 | Deed | Bead | High | Initial | Place |
| **15** | 16 | Fail | Veil | High | Initial | Voice |
| **17** | 19 | Cut | Gut | High | Initial | Voice |
| **29** | 43 | Meat | Neat | High | Initial | Voice |
| **16** | 18 | Nod | Don | High | Metathetic | Manner |
| **34** | 57 | Lean | Kneel | High | Metathetic | Manner |
| **9** | 9 | Tape | Pate | High | Metathetic | Place |
| **12** | 12 | Moan | Gnome | High | Metathetic | Place |
| **14** | 15 | Toad | Dote | High | Metathetic | Voice |
| **36** | 72 | Debt | Ted | High | Metathetic | Voice |
| **5** | 5 | Rice | Write | Low | Final | Manner |
| **22** | 26 | Hen | Head | Low | Final | Manner |
| **32** | 50 | Robe | Road | Low | Final | Place |
| **2** | 2 | Leave | Leaf | Low | Final | Voice |
| **18** | 20 | Live | Life | Low | Final | Voice |
| **30** | 47 | Fang | Fan | Low | Final | Voice |
| **10** | 10 | Nip | Lip | Low | Initial | Manner |
| **25** | 31 | Mat | Bat | Low | Initial | Manner |
| **13** | 13 | Feed | Seed | Low | Initial | Place |
| **19** | 22 | Down | Gown | Low | Initial | Place |
| **1** | 1 | Pill | Bill | Low | Initial | Voice |
| **27** | 34 | Dale | Tale | Low | Initial | Voice |
| **6** | 6 | Mob | Bomb | Low | Metathetic | Manner |
| **31** | 48 | Nail | Lane | Low | Metathetic | Manner |
| **3** | 3 | Pat | Tap | Low | Metathetic | Place |
| **21** | 24 | Dab | Bad | Low | Metathetic | Place |
| **33** | 54 | Tuck | Cut | Low | Metathetic | Place |
| **35** | 66 | Mane | Name | Low | Metathetic | Place |

The table gives the 36 pairs that were used in the present study. *Frequency* of the target (compared with the distractor) was manipulated in the original PALPA-3: for half of the items. the target has a higher frequency than the distractor; for the other half the target is lower or equivalent in frequency to the distractor. *Location* refers to the fact that pairs differ either in the initial or final positions of pairs, or in pairs that are metathetically related (i.e. the order of sounds is reversed). *Type* indicates whether the foil minimally deviates from the target in terms of voice, manner, or place of articulation.

**Table S3.** Comparison of original ANCOVA and adjusted model with relaxed normality assumptions

|  | Coefficient | Original ANCOVA  (95% CIs) | Adjusted ANCOVA (95% CIs) |
| --- | --- | --- | --- |
| lvPPA vs nfvPPA | **-2.73** | -3.80 to -1.66 | -4.60 to -1.10 |
| lvPPA vs svPPA | **-2.80** | -3.99 to -1.61 | -4.48 to -1.39 |
| lvPPA vs tAD | -0.96 | -2.18 to 0.26 | -2.77 to 0.54 |
| lvPPA vs Control | **-2.29** | -3.42 to -1.15 | -3.96 to -0.93 |
| tAD vs nfvPPA | **-1.77** | -2.97 to -0.56 | -2.98 to -0.48 |
| tAD vs svPPA | **-1.84** | -3.06 to -0.62 | -2.89 to -0.77 |
| tAD vs Control | **-1.33** | -3.82 to -0.27 | -2.27 to -0.18 |
| nfvPPA vs svPPA | -0.07 | -1.19 to 1.04 | -1.18 to 1.07 |
| nfvPPA vs Control | -0.44 | -0.60 to 1.48 | -0.68 to 1.80 |
| svPPA vs Control | -0.51 | -1.49 to 0.46 | -1.51 to 0.25 |

The main manuscript reports results from a parametric ANCOVA model. This table shows 95% confidence intervals (CIs) for between-group comparisons for the conventional ANCOVA approach, compared to non-parametric bias corrected and accelerated bootstrap confidence intervals for the between-group differences based on 10000 bootstrap resamples, relaxing assumptions of normality and homoscedasticity. Results from this more conservative approach were very similar to those using the conventional ANCOVA, and in particular the same significant group differences (in bold) were yielded using both approaches.

**Figure S1.** Neuroanatomical region of interest specified for VBM analysis

**
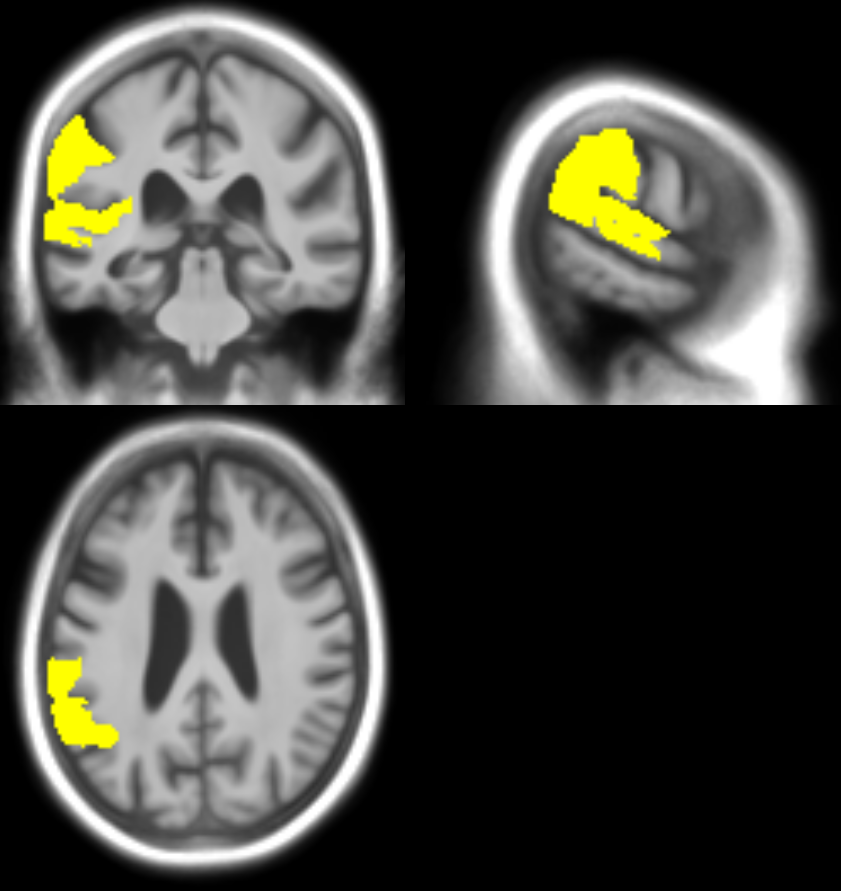
**

Representative coronal (top left), sagittal (top right) and axial (bottom) T1-weighted MRI brain sections showing the neuroanatomical region (delineated in yellow) used to correct for multiple voxel-wise comparisons in the voxel-based morphometric (VBM) analysis, based on prior anatomical hypotheses (see text). This region comprised posterior superior temporal gyrus, supramarginal gyrus, angular gyrus, and planum temporale, all in the left hemisphere.

**SUPPLEMENTARY REFERENCES**

1. Nelson H. *National Adult Reading Test (NART): For the Assessment of Premorbid Intelligence in Patients with Dementia Test Manual*. Windsor, UK: NFER-Nelson; 1982.

2. Schonell F. *Graded Word Reading Test*. Oliver & Boyd; 1900.

3. Gorno-Tempini ML, Hillis AE, Weintraub S, et al. Classification of primary progressive aphasia and its variants. *Neurology*. 2011;76(11):1006-1014. doi:10.1212/WNL.0b013e31821103e6

4. Dubois B, Feldman HH, Jacova C, et al. Revising the definition of Alzheimer’s disease: a new lexicon. *Lancet Neurol*. 2010;9(11):1118-1127. doi:10.1016/S1474-4422(10)70223-4

5. *Recommended Procedure Pure-Tone Air-Conduction and Bone-Conduction Threshold Audiometry with and without Masking*.; 2018. www.thebsa.org.uk. Accessed January 14, 2020.

6. Ridgway GR, Omar R, Ourselin S, Hill DLG, Warren JD, Fox NC. Issues with threshold masking in voxel-based morphometry of atrophied brains. *Neuroimage*. 2009;44(1):99-111. doi:10.1016/j.neuroimage.2008.08.045

7. Freedman D, Lane D. A Nonstochastic Interpretation of Reported Significance Levels. *J Bus Econ Stat*. 1983;1(4):292-298. doi:10.1080/07350015.1983.10509354

8. Efron B, Tibshirani R. *An Introduction to the Bootstrap*. Chapman & Hall; 1994.
